# Application of DNA Aptamers and Quantum Dots to Lateral Flow Test Strips for Detection of Foodborne Pathogens with Improved Sensitivity *versus* Colloidal Gold

**DOI:** 10.3390/pathogens3020341

**Published:** 2014-04-10

**Authors:** John G. Bruno

**Affiliations:** Operational Technologies Corporation, 4100 N.W. Loop 410, Suite 230, San Antonio, TX 78229, USA; E-Mail: john.bruno@otcorp.com; Tel.: +1-210-731-0015; Fax: +1-210-731-0041

**Keywords:** aptamer, colloidal gold, detergent, digoxigenin, *E. coli*, lateral flow test strip, *Listeria*, quantum dot, *Salmonella*, SELEX

## Abstract

Preliminary studies aimed at improving the sensitivity of foodborne pathogen detection via lateral flow (LF) test strips by use of high affinity DNA aptamers for capture and reporter functions when coupled to red-emitting quantum dots (Qdot 655) are reported. A variety of DNA aptamers developed against *Escherichia coli*, *Listeria monocytogenes*, and *Salmonella enterica* were paired in capture and reporter combinations to determine which yielded the strongest detection of their cognate bacteria using a colloidal gold screening system. Several promising sandwich combinations were identified for each of the three bacterial LF strip systems. The best *E. coli* aptamer-LF system was further studied and yielded a visible limit of detection (LOD) of ~3,000 *E. coli* 8739 and ~6,000 *E. coli* O157:H7 in buffer. These LODs were reduced to ~300–600 bacterial cells per test respectively by switching to a Qdot 655 aptamer-LF system. Novel aspects of these assays such as the use of high levels of detergents to avoid quantum dot agglutination and enhance migration in analytical membranes, identification of optimal analytical membrane types, UV-immobilization of capture aptamers, and novel dual biotin/digoxigenin-end labeled aptamer streptavidin-colloidal gold or -Qdot 655 conjugates plus anti-digoxigenin antibody control lines are also discussed. In general, this work provides proof-of-principle for highly sensitive aptamer-Qdot LF strip assays for rapid foodborne pathogen detection.

## 1. Introduction

The popularity of lateral flow (LF) or immunochromatographic (IC) test strips especially for common tests such as pregnancy, drugs of abuse, and food safety assessments is undeniable and driven by their ease of use, speed and relative accuracy of these simple and inexpensive disposable devices. Unfortunately, in general, standard colloidal gold or latex particle immuno-LF test strip sensitivity is limited to approximately 2,000 pathogenic *E. coli* cells per milliliter [1,2], but the food safety testing industry demands zero tolerance of foodborne pathogens (*i.e.*, no detectable pathogens). Therefore, LF strip use in food safety testing often depends on enrichment culturing to amplify pathogen numbers to detectable levels which delays screening and requires lengthy and expensive cold storage of many food products that could otherwise be sold quickly. The food industry clearly would like more sensitive test results to enable more rapid (10–20 min) decisions to clear foods for sale. Thus, ultrasensitive LF test strip development with or without an optical reader instrument is a worthwhile research and development goal.

The present report attempts to evaluate many of the existing proprietary (patented or patent-pending) foodborne pathogen DNA aptamer sequences developed by the author’s group and other investigators [3,4,5,6,7,8,9,10,11] as possible replacements for conventional antibodies to perhaps enhance sensitivity and decrease lot-to-lot reagent variability. The chosen *E. coli* aptamer DNA sequences (EcO 3R and EcO 4F) on which the present work focused are given in the Experimental Section 3.1. Other promising aptamer DNA sequences illustrated by their performance in LF screening experiments presented herein can be found in U.S. patent application number 13/136,820. As soon as a useful aptamer’s DNA sequence is known, it can be reproduced with very high fidelity by chemical synthesis [12] while comparable polyclonal antibodies are potentially subject to some degree of batch-to-batch variation [13,14]. Even comparable monoclonal antibodies are potentially subject to decreased production or “drift” and alterations in amino acid composition over long periods of time [13,14] because they are produced in hybridoma cell lines which may experience altered culture conditions or mutations over time leading to variable antibody output or composition. Due to the greater fidelity of aptamer synthesis and potentially greater affinity [12,15] of some aptamers *versus* comparable antibodies, it made sense to investigate the use of aptamers in place of antibodies in LF test strips for potentially improved foodborne pathogen detection as presented here.

Another major means to possibly improve LF sensitivity is by use of fluorescent tags [16] such as fluorescent nanoparticles (FNPs) [17], quantum dots (Qdots) [18,19,20,21,22], or upconverting phosphors [23]. The use of fluorescent particles has been demonstrated to improve LF test strip sensitivity by as much as 300-fold in some cases [17], but more commonly by at least ten-fold [20], which still represents a significant improvement in sensitivity. In general, published assessment of fluorescent LF test strip performance has been visual. However, the use of quantitative fluorescence instrumentation and objective analysis of signal-to-noise ratios may enhance sensitivity further. And signal-to-noise ratios can also be enhanced by time-resolved fluorometry [17], but such time-resolved technology is not widespread or affordable for many laboratories. Hence, herein the simple use of several extant and independently validated foodborne pathogen aptamers [10,24] in LF test strips are illustrated and in the case of general *E. coli* detection, a comparison is made between colloidal gold and Qdot versions of a preliminary LF assay at its approximate limit of detection (LOD).

## 2. Results and Discussion

### 2.1. Solving the Little Known Problems of Aptamer and Qdot Use in LF Test Strips

#### 2.1.1. Immobilizing Capture Aptamers and Obtaining Intense Control Lines

**Figure 1:**
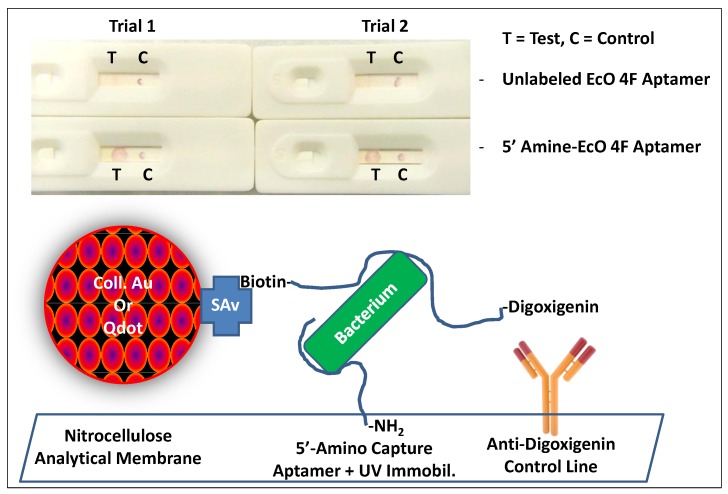
**Top:** Comparison of two lateral flow (LF) test strip trials illustrating that a 5ʹ-primary amine-C6 linker EcO 4F capture aptamer and UV light were required to immobilize the capture aptamer, because no detection of *E. coli* 8739 was seen in trials in which UV light was not first used or the capture aptamer was not amine end-labeled (not shown). EcO 3R-colloidal gold was used as the reporter conjugate. **Bottom:** The general LF test strip scheme involving digoxigenin-5ʹ-reporter aptamer-3ʹ-biotin-streptavidin-colloidal gold or –Qdot conjugates, amino-capture aptamers immobilized with UV light on the test (T) line or dot and air-dried anti-digoxigenin antibody on the control (C) line or dot.

Capture DNA aptamers cannot simply be air dried onto nitrocellulose or other analytical membranes since they may wash out with the advancing liquid front during sample wicking. It is well-known that nucleic acids including aptamers can be covalently bonded to nitrocellulose via ultraviolet light [25]. Thus, 15 min exposure to 254 nm ultraviolet (UV) light was attempted for immobilization of an unlabeled and a 5ʹ-primary amine-C6-labeled version of the *E. coli* aptamer EcO 4F previously developed by the author’s laboratory [4] and independently validated by another laboratory for use in an electrochemical biosensor [24]. Figure 1 (top panel) demonstrates that only the 5ʹ-amino-labeled version of the Eco 4F capture aptamer was able to capture *E. coli* 8739 bacteria (~10^4^ live cells per test) as evidenced by the pink to purple test (T) dots in both trials where UV light was applied. One microliter “dots” were typically applied to the membranes, for test (T) and control (C) lines since it was difficult to make consistent lines with a pipettor. Therefore, it became apparent that UV light must be used in conjunction with amino end-labeled aptamers to achieve covalent capture aptamer immobilization on nitrocellulose analytical membranes. The unlabeled capture aptamers were either washing out of the membrane during sample processing or they were being immobilized in a flat configuration on the membrane. The end-labeled amino group may be providing a useful tethering point from which the rest of the aptamer can stand up vertically away from the membrane’s surface and capture bacteria as they transit through the capture dot or line. Regardless of the actual molecular configuration, it was clear that the terminal amino group and UV light were necessary for successful capture of *E. coli* at least in the LF system depicted in Figure 1 (bottom panel). It should be noted that immobilization of capture aptamers using a 254 nm UV light could also induce structural changes to the aptamers by causing thymine dimerization. Therefore, aptamers that might work in other assay formats may not work in LF test strips or their performance may be altered by UV immobilization.

Another little known problem or deficiency of aptamer-based LF test strips, is the production of a strong and reliable control line. In the past, partial hybridization of an aptamer’s primer region with the cDNA primer or a probe region has been reported or theorized to capture the colloidal gold or other conjugate aptamer-coated particles as they transit the control dot or line [26,27]. Unfortunately, this approach did not work well in any of the preceding aptamer-LF development experiments leading up to this report [28]. Hence another approach involving a 5’-biotin-aptamer-3’-digoxigenin (dual-labeled) aptamer designated EcO 3R [4,8,24] bound to streptavidin-colloidal gold or streptavidin-Qdots and an anti-digoxigenin antibody control (C) line was attempted as illustrated in Figure 1 (bottom). Figure 1 (top panel) and several of the other figures in this report show that this digoxigenin end-labeled aptamer plus anti-digoxigenin antibody control line approach consistently produces strong control (C) lines or dots.

#### 2.1.2. Sandwich Pair Screening and Choice of Optimal Analytical Membrane

**Figure 2:**
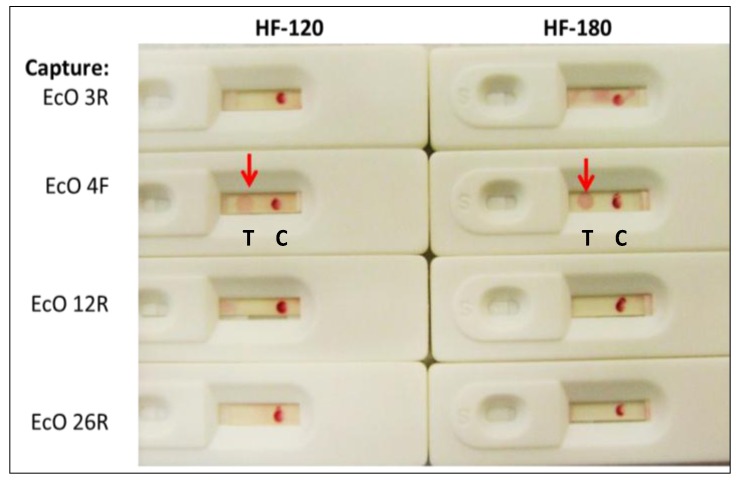
Results of the general *E. coli* (ATCC 8739 or Crook’s strain) LF test strip screening experiment with ~10^4^ live *E. coli* per LF test led to selection of 5ʹ-biotin-EcO 3R-3ʹ-digoxigenin on streptavidin-40 nm colloidal gold (used for all LF strips in this figure) and EcO 4F-5ʹ-amine capture aptamer dot on HF-180 membranes as the best system to pursue further (red arrows). Notice also the strong anti-digoxigenin control (C) lines or dots in each case.

Figure 2 illustrates that despite strong performances in ELISA-like miroplate assays (ELASA) [4], not all candidate *E. coli* aptamer conjugate/capture pairings worked in the LF format to produce visibly positive test dots (T) using ~10^4^ live *E. coli* 8739 cells per test. The LF screening shown in Figure 2 led to selection of EcO 3R and 4F as the best reporter conjugate/capture aptamer combination for further LF development. In addition, Millipore High Flow (HF)-180 proved to be the optimal analytical membrane *vs*. HF-120 and other faster membranes as shown by the red arrows in Figure 2. This is presumably because migration time is longer (180 *vs*. 120 s), thereby allowing greater time for bacterial capture by the amino-C6-EcO 4F capture aptamer. HF-240 (not shown) produced even stronger capture dots, but the colloidal gold conjugate did not always migrate completely to the end in the slower, smaller pore size, HF-240 membranes. These same EcO *E. coli* aptamers have also demonstrated utility in published competitive fluorescence assays [4], aptamer-magnetic bead capture assays [8], and electrochemical biosensors [24] in the hands of other researchers, thereby further validating them as quality reagents for general *E. coli* LF test strip development. The actual DNA sequences of these aptamers are reported in Section 3.1. All aptamer sequences used in this work are proprietary and patented or patent-pending in all cases.

**Figure 3:**
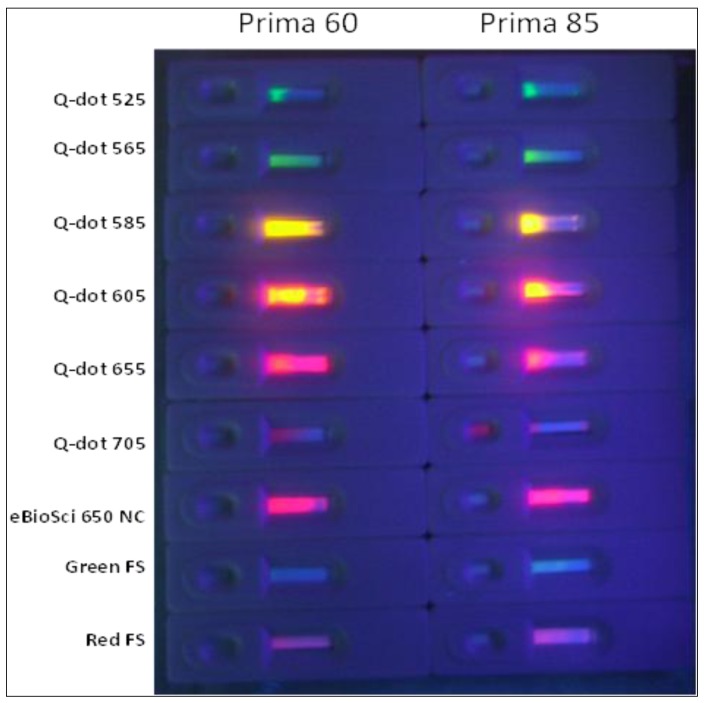
The problem of poor Qdot and nanocrystals (NC) or Fluospheres (FS) migration in LF test strips. Various sizes (colors) of commercially available Qdots up to ~6 nm in diameter [29] should be able to migrate freely through most nitrocellulose analytical membranes, but they clearly cannot, perhaps due to aggregation of individual Qdots and FNPs into much larger agglomerates. See the Experimental Section for commercial sources of all particles.

2.1.3 Prevention of Qdot Aggregation and Poor Migration via High Levels of Detergents

Another major question to answer and obstacle to overcome in preliminary work was the lack of good mobility of Qdots in nitrocellulose analytical membranes despite their very small size (up to 5–6 nm for red Qdots [29]). Figure 3 illustrates the putative Qdot aggregation and mobility problem with a variety of different colored (different sized) Qdots and plastic fluorophore-doped nanoparticles (FNPs) from various commercial sources without aptamer coatings. None of these particles migrated very far or completely through even the largest pore-size (fastest) analytical membranes such as Whatman Prima 60 and 85 membranes (Figure 3). This problem strongly suggested agglutination of the Qdots and other FNPs. This migration problem has not been acknowledged in most Qdot immunochromatographic or LF test strip journal articles with the exception of Berlina, *et al*. [21,22] who acknowledged the migration problem and revealed the use of 5% Tween 20 and a few other detergents as a solution to counteract Qdot clumping. The author was apprehensive about the effect of adding such a high level of detergent (5%) on aptamer binding efficacy, but began adding 5% Tween 20 to the stock red Qdot 655-streptavidin-5ʹ-biotin-EcO 3R-3ʹ-digoxigenin reagent which produced a much more mobile Qdot-aptamer conjugate as seen later in this report. 

**Figure 4:**
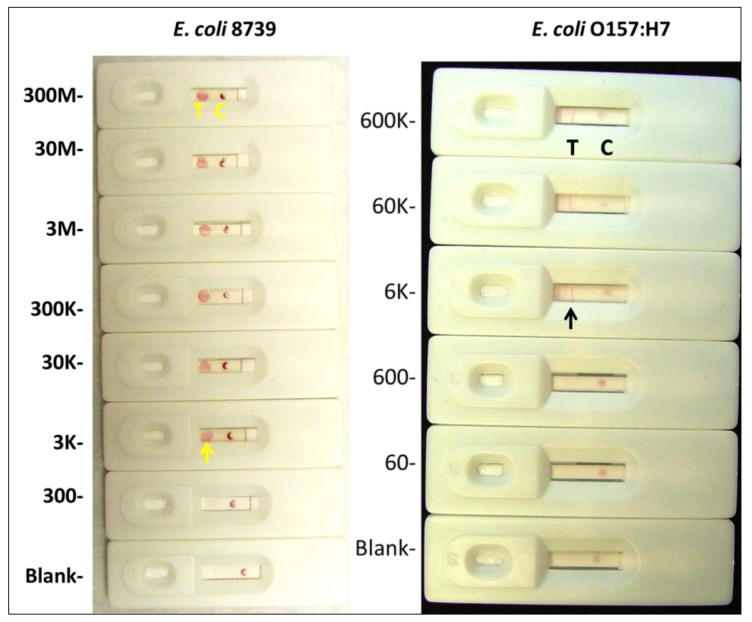
Representative results of several sensitivity tests using live *E. coli* cells demonstrates a limit of detection (LOD) of ≤ 3,000 cfu for the 8739 strain and ≤ 6,000 colony forming units (cfu) for *E. coli* O157:H7 using visual detection of colloidal gold.

#### 2.1.4. Visual Determination of LOD for Colloidal Gold and Qdot Versions of the *E. coli* LF Assay

Figure 4 (left side) illustrates that the approximate visual detection limit for the 8739 (Crooks) strain of *E. coli* was ~3,000 live cells per test. The LOD for a relevant foodborne pathogenic strain of *E. coli* such as O157:H7 was found to be about twice as high at approximately 6,000 live cells per test (Figure 4, right side). Figure 4 presents representative results of several trials of the titration experiments using the Eco 3R-colloidal gold conjugate and Eco 4F-5ʹ-amino capture LF system. Reasons for the differences in LOD between the 8739 and O157:H7 strains of *E. coli* are not known at present, because the EcO 3R and 4F aptamer targets on the surface of *E. coli* are not defined. It seems likely that these aptamers bind to an outer membrane protein (OMP) or perhaps a relatively common epitope on lipopolysaccharide (LPS) which may vary in amount per cell or composition between strains, thereby accounting for the differences in LOD.

**Figure 5:**
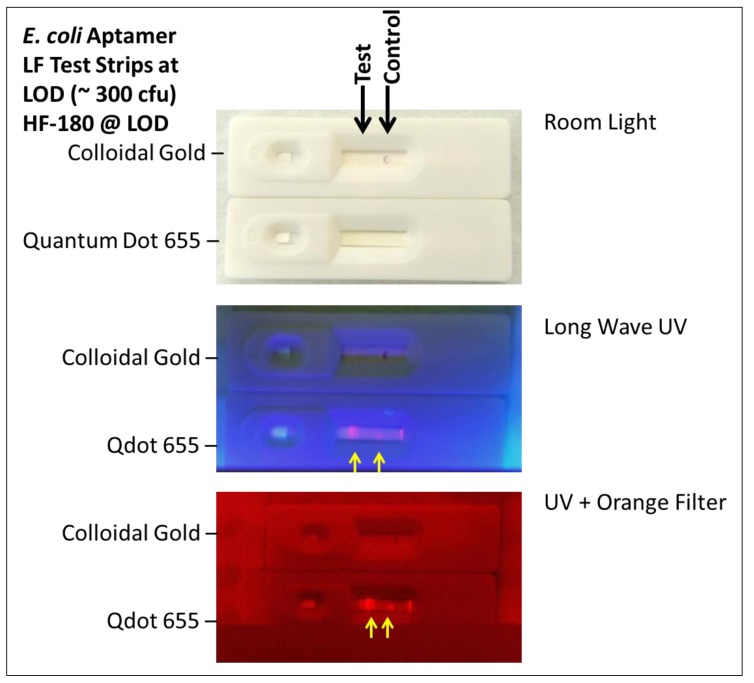
Comparison of a Qdot 655 version of the EcO 3R/4F aptamer test strip with the conventional colloidal gold version of the same strip at the colloidal gold LOD of ~300 *E. coli* 8739 cell per test showing at least 10 times greater sensitivity with Qdots and a handheld long wave UV light and visual detection *versus* colloidal gold. The capture (test or T) line or dot is clearly visible with Qdots, but not with colloidal gold at this low level of bacteria (300 cfu or viable cells).

Figure 5 illustrates, that the use of Eco 3R aptamer-Qdot 655 conjugate appears to enhance the LOD at least ten fold to 300 live *E. coli* 8739 cells or colony forming units (cfu) per test. In particular, the colloidal gold capture dot is not visible to the naked eye in any of the panels of Figure 5, but under a long wavelength (365 nm) UV lamp, the pink Q dot line is visible and can be enhanced by means of an orange glass filter as shown in the bottom panel of Figure 5. The ten-fold increase in sensitivity is consistent with other reports in the literature for antibody-based test strips [20]. Comparison of results from Figure 3 and Figure 5 also illustrates the very positive impact of 5% Tween 20 addition on the migration of red Qdots in HF-180 membranes as per Berlina, *et al*. [21,22].

#### 2.1.5. Cross-Reactivity Assessment of the *E. coli* Aptamer LF Test

**Figure 6:**
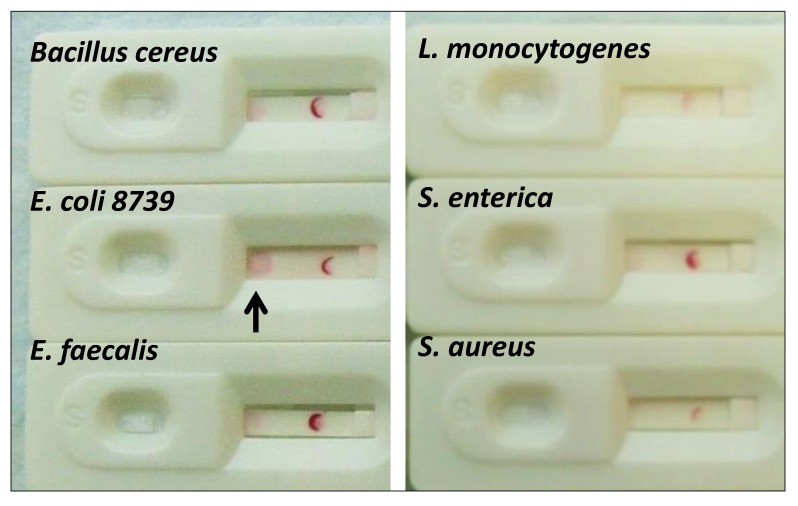
Results of a preliminary EcO 3R/4F LF cross-reactivity study showing the darkest capture dot for *E. coli* 8739 with minimal reactivity for *Bacillus cereus*, *Enterococcus faecalis*, *Listeria monocytogenes*, *Salmonella enterica* serovar Typhimurium and *Staphylococcus aureus* all at ~10^4^ cfu per test. Digoxigenin control lines were used on each strip.

Preliminary cross-reactivity or specificity studies were conducted as well for the *E. coli* system (*i.e.*, EcO 3R/4F) as illustrated in Figure 6. The results were encouraging and suggested good specificity to the level of detecting *E. coli* (strain 8739 and O157:H7 to a lesser extent) *versus* several other species as indicated in Figure 6.

#### 2.1.6. Screening for Other Potential Foodborne Pathogen LF Assays

**Figure 7:**
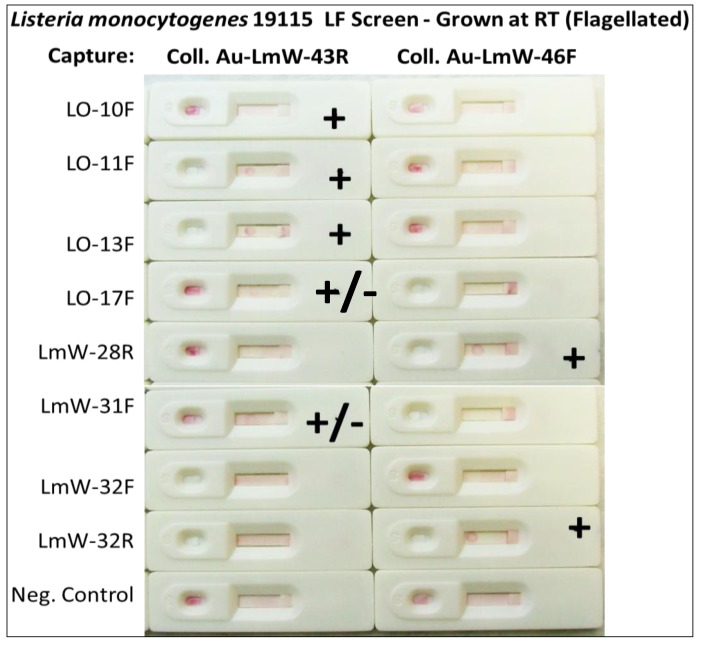
Results of preliminary *L. monocytogenes* strain 19115 aptamer LF colloidal gold screening without control lines or dots. Promising sandwich combinations are shown with a + or +/− (faint or ambiguous) following addition of ~10^4^ live cells per test.

**Figure 8:**
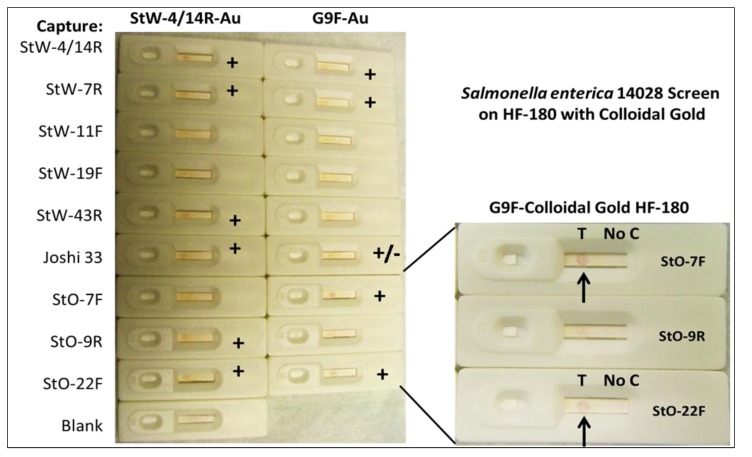
Results of preliminary *Salmonella enterica* strain 14028 aptamer LF colloidal gold screening without control lines or dots. Promising combinations are shown with an arrow following addition of ~10^4^ cells per test. Some of the better combinations are magnified in the lower right inset. One *Salmonella* aptamer sandwich combination employing an aptamer from Joshi *et al*. [10] also appeared promising.

Figure 7 and Figure 8 demonstrate that other sandwich aptamer pairs from extant pools [8,10] can be identified for successful aptamer-LF test development to detect *Listeria monocytogenes* and *Salmonella enterica* (serovar Typhimurium, formerly *S. typhimurium*). All capture aptamers in both Figure 7 and Figure 8 were 5ʹ-amino labeled and UV-immobilized, but no anti-digoxigenin control lines or dots were added for screening. All screening experiments were performed with ~10^4^ live cells per test. Interestingly, combinations which worked best in aptamer-magnetic bead-based assays developed and published by the author’s laboratory [8] frequently worked well in the LF sandwich assay format as well as denoted by plus (+) marks in Figure 7 and Figure 8 while faint or ambiguous test dots were denoted by +/− symbols. Of note in Figure 7, aptamers designated by the prefix “LO” were developed against the listeriolysin O protein [8]. Likewise aptamers bearing the “LmW” prefix were developed against whole live and unfixed *L. monocytogenes* cells and tended to detect *L. monocytogenes* grown at room temperature (~25 °C). Growth at room temperature enables expression of flagella from *L. monocytogenes* and suggests that these aptamers bind *Listeria* flagellins. 

Similarly in Figure 8, some sandwich aptamer pairings yielded encouraging positive capture dots indicated by plus signs. Some of these aptamers were developed against *Salmonella* whole cells (StW prefix) and some were developed against chaotrope-extracted (1.5M MgCl_2_) [6] outer membrane proteins (StO prefix). The author also included a 5ʹ-amino capture aptamer sequence against *Salmonella* published by Joshi *et al*. [10], designated “Joshi 33” which gave a positive or faintly positive test dot when paired with two of the author’s *Salmonella* capture aptamer-colloidal gold conjugates as seen in Figure 8.

## 3. Experimental Section

### 3.1. Preparation of Aptamer-Colloidal Gold and Qdot Conjugates

Digoxigenin was added to the 3ʹ end of 5ʹ-biotinylated DNA aptamers during chemical synthesis at Integrated DNA Technologies (Coralville, IA, USA), but this process can require truncation of a few terminal bases from the constant primer region to accommodate the 3ʹ digoxigenin, because supporting the oligonucleotide during synthesis on a solid support is problematic for longer oligonucleotides having 3ʹ modifications (personal communication with Integrated DNA Technologies). Alternatively, although not performed during the reported work, commercially available N-hydroxysuccinimide (NHS)-ester digoxigenin (available from Sigma-Aldrich and other sources) can be added to 3ʹ-amino-terminated 5ʹ-biotinylated aptamers, followed by purification in the void volume of a Sephadex G25 (PD-10, GE Healthcare, Inc., Piscataway, NJ, USA) column. For this work, 500 µL of 0.1 to 0.12 mg/mL 3ʹ-digoxigenin-aptamer-5ʹ-biotion was mixed with 500 µL of 10 O.D. streptavidin-40 nm colloidal gold from DCN (Diagnostic Consulting Network, Carlsbad, CA, Product No. PACG-060) or 50 µL of streptavidin-Qdot 655 reagent (Life Technologies, Cat. No. Q10123MP, having 5–10 streptavidin molecules per Qdot) in sterile phosphate buffered saline (PBS, pH 7.2) for 1 hour at room temperature. Other Qdots and FNPs for the mobility tests were also obtained from Life Technologies, Inc. (Carlsbad, CA, USA) or eBioScience/Affymetrix (San Diego, CA, USA). The reporter conjugates were then split into equal aliquots and purified by separation from excess digoxigenin-aptamer-biotin molecules using 30 kD molecular weight cut off (MWCO) spin columns (Millipore Amicon Ultracel^®^). The columns were spun for 2 min at 14,500 rpm (14,000× G) in an Eppendorf MiniSpin^®^ Plus microcentrifuge and the eluates were discarded. The retained conjugates were then washed twice in 500 µL of sterile PBS each time with spinning for 2 min per wash as before to ensure purity. The reporter conjugates were then reconstituted in 500 µL of sterile PBS and stored at 4 °C until used in LF tests. Tween 20 was added at 5% as per Berlina *et al*. [21,22] to the Qdot-aptamer conjugates to prevent putative agglutination. 

The two main *E. coli* aptamers on which the present work focused were as follows:

**EcO 3R** (truncated to 60 bases for 3ʹ-digoxigenin modification):

CACACCTGCTCTGTCTGCGAGCGGGGCGCGGGCCCGGCGGGGGATGCGTGGTGTTGGCTC

**EcO 4F** (73 base length):

ATACGGGAGCCAACACCATAATATGCCGTAAGGAGAGGCCTGTTGGGAGCGCCGTAGAGCAGGTGTGACGGAT

The flanking 18 base forward and reverse primer regions, or what remained of them after truncation, are underlined in the above listing. The primer regions were included in each of the aptamers used in this study. DNA sequences of other promising aptamer pairs described herein can be found in U.S. patent application number 13/136,820.

### 3.2. Assembly and Preparation of LF Test Strips

Millipore High Flow^®^ (HF) or Whatman Prima^®^ nitrocellulose analytical membrane strips (4 cm in length) of various grades or porosities as noted in the results section (up to HF 240) were gently pressed onto the middle region of adhesive 6 cm plastic laminate backing strips with gloved hands. Whatman Standard 17 conjugate pad strips were then laid onto the adhesive at the bottom of the analytical membrane with 4 mm overlap onto the analytical membrane and Whatman GB002 sample pad strips with a 4 mm overlap onto the conjugate pad. Finally, Whatman 470 wicking pad strips were laid onto the adhesive laminate at the top of the analytical membrane with 4 mm overlap and 4 mm LF test strips were cut with a paper cutter. One microliter of stock 5ʹ-amino-C6 to C12 linker-aptamers at ~1.5 µg/mL for capture were applied on the first half of HF nitrocellulose analytical membranes in each case and air dried for 10 min. LF strips were then placed in plastic cassette bottoms (DCN, MICA-125) and UV baked for 15 min in a UVP CL-1000 oven (254 nm emission wavelength and total energy imparted ~20 milliJoules/cm^2^). One µL of 0.5 mg/mL anti-digoxigenin antibody (recombinant rabbit monoclonal antibody clone 9H27L19, Life Technologies, Inc., Cat. No. 700772) was then applied on the second half of the analytical membrane and allowed to air dry for at least 10 min at room temperature. The upper or top half of the plastic cassette (DCN, MICA-125) was then snapped onto the bottom half to complete the LF test strip housing.

### 3.3. LF Screening, LOD Titration and Cross-Reactivity Experiments

All bacterial cultures (*Bacillus cereus, Enterococcus faecalis, Escherichia coli* 8739 (Crooks strain), *E. coli* O157:H7, *Listeria monocytogenes* 19115, *Salmonella enterica* serovar Typhimurium 14028, and *Staphylococcus aureus*) were obtained from Microbiologics, Inc. (St. Cloud, MN, USA) or American Type Culture Collection (ATCC, Manassas, VA, USA) and cultured overnight on blood agar plates at 37 °C with the exception of *L. monocytogenes* which was cultured for two days at room temperature (~25 °C) to encourage expression of flagella. Five mL of sterile PBS was added to the surface of blood agar plates and bacteria were gently scraped from the surface by means of sterile plastic hockey sticks. Bacteria were siphoned from plates with a sterile pipette and diluted in 20 mL of sterile PBS. Serial ten-fold dilutions were made in sterile PBS and used to quantify bacterial concentrations by average spread plate counts performed in triplicate on blood agar plates cultured at 37 °C overnight in all cases.

Approximately 10^4^ live bacteria in 50 µL of PBS were added to the sample port of each LF test strip for screening and cross-reactivity experiments and chased with 100 µL of sterile PBS. Approximate LODs were determined from serial 10-fold dilution experiments conducted in triplicate and validated by overnight 37 °C spread plate counts of the lowest concentration of bacteria detected. All sensitivity and cross-reactivity determinations were assessed visually by the author after allowing LF test strips to fully develop for at least 20 min. Qdot-based LF tests were assessed in room light, followed by illumination with a longwave UV light (365 nm) in a dark room and use of an orange Schott glass filter to enhance visual detection of Qdot 655 particles.

## 4. Conclusions

In the broad view, preliminary results presented in this report, reveal that it is possible to develop sensitive aptamer-based LF test strips for foodborne pathogen detection which rival antibody-based LF strips. Although, sensitivity exceeding that of reported immuno-test strips for other pathogenic strains of *E. coli* [1,2] was not noted, the potential to far exceed antibody-based LF sensitivity may be realized by development of longer (up to 200 base) “multivalent” aptamers [30,31,32]. Longer multivalent aptamers having multiple binding sites for different epitopes should theoretically exhibit greater avidity and, perhaps, greater specificity *versus* the existing 60–73 base aptamers. My group is currently developing lengthier aptamers under a USDA grant in the hope of differentiating the various LPS molecules produced by the “Big 6” non-O157 Shiga toxin producing *E. coli* (STEC) for use in LF test strips. Even if aptamers only ever match the performance of antibodies, the reduced cost associated with obviating animals for reagent development, coupled with the greater batch-to-batch reproducibility of aptamers [12], should make them very attractive reagents for LF test strip development in the future. As previously indicated, numerous aptamers against bacteria and the biotoxins they produce have already been developed and characterized for other types of assays and applications [3,4,5,6,7,8,9,10,11]. As this work has demonstrated, these aptamer reagents can readily be adapted to LF formats and perhaps exploited for food safety applications in the near future.

Several key lessons were learned during the course of this research. For example, much stronger and more robust control lines or dots can be achieved by the use of dual biotin and digoxigenin end-labeled aptamers on streptavidin-coated colloidal gold or Qdots in conjunction with an air-dried anti-digoxigenin monoclonal antibody in the control line or dot *versus* an aptamer and primer hybridization scheme [26,27]. Clearly too, a 5ʹ- or 3ʹ-primary amine-labeled aptamer with 6 or 12 carbon length linker and 15 min UV baking of the capture line or dot on nitrocellulose is necessary for robust capture of bacteria. Amino-terminated aptamers must be UV baked onto the analytical membrane or they may wash away during wicking. In addition, Qdot conjugates probably agglutinate in the absence of high levels of detergents, making it difficult for them to migrate consistently into or through even large porosity analytical membranes. However, this problem was solved by addition of 5% Tween 20 detergent to the stock aptamer-biotin-streptavidin-Qdot suspension as suggested by Berlina, *et al*. [21,22]. Qdot conjugates having less than 5% Tween 20 did not fully migrate across the LF test strips. Millipore’s High Flow (HF)-180 with a 180 s transit time emerged as the optimal analytical membrane for its combination of speed and full mobility of colloidal gold and Qdots through the 4 cm length of the nitrocellulose membrane.

The best *E. coli* sandwich combination (EcO 3R-conjugate and EcO 4F-capture aptamer) appears to produce a visible LOD of ~3,000 live *E. coli* 8739 cells and 6,000 live *E. coli* O157:H7 cells by visible colloidal gold detection in relative agreement with previously published results for these two types of *E. coli* [4,9]. The detection limit was enhanced ten-fold to ~300 cells per LF test with Qdot 655 and UV excitation as reported by other investigators in similar Qdot-antibody LF systems [20]. Visual detection of Qdots in LF strips was accentuated by the use of a simple orange glass filter. Finally, several sandwich aptamer combinations for *Listeria monocytogenes* (listeriolysin O or flagellins and *Salmonella enterica* whole cells from existing aptamers [8,10] were identified. Clearly, all three foodborne pathogen detection systems require more development, and validation, but initial results are encouraging and point to the potential for very sensitive fluorescent aptamer-LF tests in the future. Future work will focus on expanded testing of the promising sandwich aptamer combinations identified herein against many other species and strains, as well as testing in various food matrices and enrichment culture media.
